# MEMS IMU Error Mitigation Using Rotation Modulation Technique

**DOI:** 10.3390/s16122017

**Published:** 2016-11-29

**Authors:** Shuang Du, Wei Sun, Yang Gao

**Affiliations:** 1 School of Aeronautics and Astronautics, University of Electronics Science and Technology of China, Chengdu 610000, China; 2 School of Geomatics, Liaoning Technical University, Fuxin 123000, China; sunwei-3775235@163.com; 3 Department of Geomatics Engineering, The University of Calgary, Calgary, AB T2N 1N4, Canada; ygao@ucalgary.ca

**Keywords:** MEMS IMU, error mitigation, rotation modulation, calibration

## Abstract

Micro-electro-mechanical-systems (MEMS) inertial measurement unit (IMU) outputs are corrupted by significant sensor errors. The navigation errors of a MEMS-based inertial navigation system will therefore accumulate very quickly over time. This requires aiding from other sensors such as Global Navigation Satellite Systems (GNSS). However, it will still remain a significant challenge in the presence of GNSS outages, which are typically in urban canopies. This paper proposed a rotary inertial navigation system (INS) to mitigate navigation errors caused by MEMS inertial sensor errors when external aiding information is not available. A rotary INS is an inertial navigator in which the IMU is installed on a rotation platform. Application of proper rotation schemes can effectively cancel and reduce sensor errors. A rotary INS has the potential to significantly increase the time period that INS can bridge GNSS outages and make MEMS IMU possible to maintain longer autonomous navigation performance when there is no external aiding. In this research, several IMU rotation schemes (rotation about X-, Y- and Z-axes) are analyzed to mitigate the navigation errors caused by MEMS IMU sensor errors. As the IMU rotation induces additional sensor errors, a calibration process is proposed to remove the induced errors. Tests are further conducted with two MEMS IMUs installed on a tri-axial rotation table to verify the error mitigation by IMU rotations.

## 1. Introduction

Tens of years ago, to maintain long term autonomous navigation performance, the inertial navigation system (INS) was designed based on high end inertial sensors such as ring laser gyro (RLG) and fiber optical gyro (FOG). However, the large size (usually very heavy as well) and high cost have limited their use to various applications. With the development of micro-electro-mechanical system (MEMS) technology, the MEMS-based inertial sensors became available to the commercial market in 1990s and they quickly found applications for indoor pedestrian and robotic navigation because of their low-cost, small-size, light-weight and low-power consumption [1,2]. MEMS IMU outputs, however, are corrupted with significant sensor errors, such as high frequency noise, bias, scale factors and installation errors. As a result, the navigation errors will accumulate quickly and deteriorate the navigation solution over a short time period [2,3,4,5]. For example, the position errors of a low-cost MEMS IMU will grow to kilometers within several minutes. To limit the accumulation of navigation errors in INS, aiding from other sensors is required. For example, GNSS position and velocity can be used to estimate the INS errors using an extended Kalman filter [6,7,8,9]. Various investigations have been conducted to employ external navigation information to limit the INS error accumulation. The neural networks are applied to mimic the behavior of navigation error accumulation of INS [10,11], and the fuzzy logic is also employed to adaptively adjust the parameters of the estimation algorithm [12]. The auto-regressive model was also proposed to interpret the randomness of the MEMS-based inertial sensor errors [3,13].

This paper investigates a different approach to limit the accumulation of navigation errors in MEMS-based INS. Instead of focusing on the use of external aiding information by existing methods, it uses a rotary INS to reduce the navigation error growth by rotating the IMU. A rotary INS is an inertial navigator in which the IMU is installed on a rotation platform. By rotating the IMU in certain pre-defined schemes, it can change the characteristics of the inertial sensor errors and consequently reduce the accumulation of navigation errors. For example, the rotation of IMU with a constant angular rate can modulate the constant inertial bias into periodic signals and an integration of the modulated inertial data over a complete rotation cycle can eliminate the bias impact on the navigation solutions. Rotary INS technique has been widely applied in marine navigation for submarines and warships [14,15,16]. However, only few researches have been conducted on rotary INS based on low-cost MEMS IMU, which has great potential applications in the near future and is the focus of this paper. A rotary MEMS-based INS has the potential to significantly increase the time period that it can bridge GNSS outages for GNSS/MEMS IMU integrated systems [17,18]. It also makes MEMS IMU possible to maintain much longer autonomous navigation performance as a self-contained navigation system when there is no external aiding available.

Although the requirement for a rotational platform would increase the complexity and cost of the use of the rotation modulation technique, it will be advantageous to attach the MEMS IMU to a rotating platform that is already built in the vehicle, such as the wheel for land vehicle, the airscrew for the helicopter, and the propeller for the boat, just to mention a few. In a recent research, gyros are attached to the wheel of a ground vehicle and the results have demonstrated that a low-cost MEMS IMU can provide a very accurate navigation solution using the rotation modulation technique [18]. In the near future, the advances in hardware technologies would also reduce the complexity and cost in developing rotational IMU devices, creating increased applications. In addition to the mitigation of navigation error accumulations in a non-aiding mode, the rotation of IMU will help improve the system observability as the observability of inertial sensor errors are related to IMU orientations [1]. Overall, the rotary MEMS-based inertial system is expected to significantly improve the navigation performance comparing to a non-rotary one.

Comparing to the low-cost MEMS IMU, FOG and RLG errors are orders of magnitude smaller, and thus the application of the rotation modulation technique to FOG and RLG based IMU can effectively mitigate the error accumulations. However for MEMS IMU, due to their significant bias instability, scale factor, installation errors, as well as noise, how can the application of the rotation modulation technique help reduce the navigation errors is not clear so far which requires investigation. Although the idea of MEMS-based rotary INS had already been proposed, it was verified only by simulations in previous research [17]. Real rotated tests must be conducted in order to fully investigate the feasibility of MEMS-based rotary systems for practical applications. This is the main objective of this contribution with the following specific research efforts:

(1) Error analysis for IMU rotation about X- and Y-axes: As previous research indicated that the IMU rotation about the Z-axis cannot modulate the errors in the Z-axis, which results in accumulated azimuth and velocity errors, this research conducts error analysis with IMU rotations about the X- and Y-axes, and compares three different rotation schemes for navigation applications.
(2) The calibration of gyro scale factor and installation errors: As the gyro scale factor and installation errors induce additional sensor errors when IMU rotates, which will deteriorate the error migration performance, a process to calibrate gyro scale factor and installation errors is proposed for the MEMS-based inertial system.
(3) Experiments and tests with real rotated data: As MEMS IMU outputs contain significant bias instability error, the sensor biases will vary randomly over time, not behave as constants. Tests with real rotated data are necessary in order to study the feasibility of MEMS-based rotary inertial system in real scenario.

The remainder of this paper is organized as follows. Section 2 describes the rotary INS, including the mechanization algorithm and the error mitigations with different IMU rotation schemes, each rotating about X-, Y- and Z-axes, respectively. The related mathematical equations are also derived and provided in this section. As the IMU rotation induces additional sensor errors, a calibration process is proposed and introduced in Section 3. The tests of two different MEMS IMUs on a tri-axial rotation table are given to verify the proposed calibration algorithm and error mitigation performance using rotary INS in Section 4. The conclusions and future work are summarized in Section 5.

## 2. Rotary Inertial Navigation System

**Figure 1:**
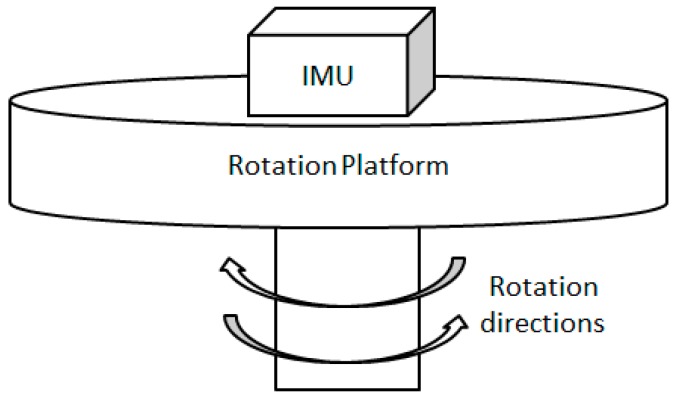
Structure of rotary inertial navigation system (INS).

The concept of rotary INS was initially proposed for the gimbaled inertial system by [19]. Afterwards, this technique was applied to a strapdown inertial system [19,20]. As certain rotations of IMU can mitigate the INS navigation errors without the requirement of external information, the rotary INS was extensively employed for the warship or submarine. The rotary INS normally contains two components, an IMU and a rotational platform, as shown in Figure 1. In the sequel, INS with non-rotating IMU is referred to as conventional INS to distinguish it from rotary INS.

### 2.1. Rotary INS Mechanization

As the IMU is rotating in the rotary INS, a new frame in which the inertial readings are collected is introduced, in addition to the traditional coordinate frames in conventional INS. This new coordinate frame can be referred as inertial sensor frame or IMU frame, and its axis are aligned with the sensitive axis of inertial sensors with the origin defined as the origin of IMU.

Generally speaking, the mechanization algorithm of rotary INS is very similar to the one of conventional INS. As the inertial readings are collected in sensor frame, a transformation process is required, as shown in Equations (1) and (2).

(1) $$f_{ib}^{b}=C_{s}^{b}(f_{is}^{s}+f_{sb}^{s})$$

(2) $$\omega_{ib}^{b}=C_{s}^{b}(\omega_{is}^{s}+\omega_{sb}^{s})$$

where $f_{ib}^{b}$ and $\omega_{ib}^{b}$ are the specific force and angular rate in body frame with respect to inertial frame, respectively; $C_{s}^{b}$ is the transformation matrix from senor frame to body frame; $f_{is}^{s}$ and $\omega_{is}^{s}$ are the specific force and angular rate in sensor frame with respect to inertial frame, respectively; and $f_{sb}^{s}$ and $\omega_{sb}^{s}$ are the specific force and angular rate of body frame with respect to sensor frame; respectively. As the rotation of IMU does not introduce any linear movement, $f_{sb}^{s}$ is a zero vector, while $\omega_{sb}^{s}$ is related to the IMU rotation schemes.

**Figure 2:**
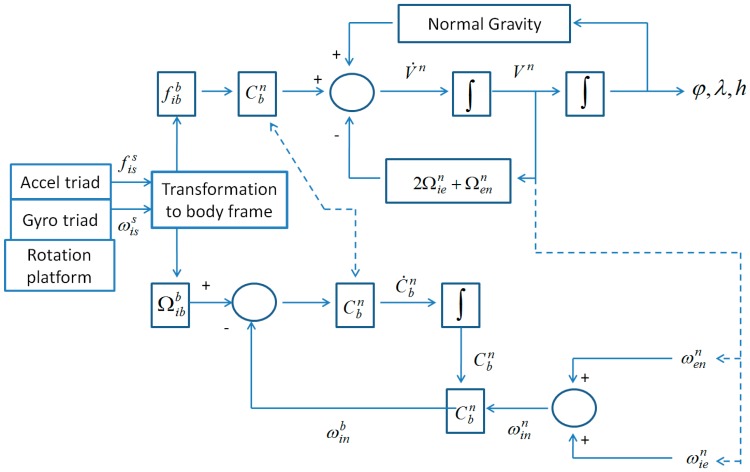
Flowchart of rotary INS mechanization.

With the transformed specific force and angular rate in body frame, the mechanization of the conventional INS can be used to derive position, velocity and attitude solutions as shown in Figure 2, where $C_{b}^{n}$ represents the transformation matrix from the body frame to the navigation frame, n represents the navigation frame, Ω represents the skew-symmetric matrix of angular rate ω, V represents the velocity, and φ,λ,h represent the latitude, longitude, and height, respectively. Apparently, the rotation angle between body frame and sensor frame is required in the transformation process, and this is usually measured by a device installed in rotation platform.

### 2.2. Error Mitigations with Different IMU Rotations

The inertial sensor errors are firstly introduced and the mitigation of navigation errors is then investigated using three different IMU rotation schemes, each rotating about X-, Y- and Z-axes, respectively. All analyses are conducted in static mode and the body frame is assumed to be aligned with the navigation frame to simplify the analysis.

#### 2.2.1. Inertial Sensor Errors

The specific forces and angular rates collected by the accelerometer and gyro triads contain different types of errors, such as noise, turn on biases, scale factor errors and installation errors. The sensor errors are the major error source that causes navigation errors. Usually, the sensor error model for gyros and accelerometers can be described by Equations (3) and (4), respectively [21].

(3) $$\tilde{\omega }=\omega +d+S_{g}\omega +N_{g}\omega +n_{w}$$

(4) $$\tilde{f}=f+b+S_{a}f+N_{a}f+n_{f}$$

where $\tilde{\omega }$ is the actual gyro outputs, ω is the true gyro angular rate, d is the gyro bias, $S_{g}$ is the scale factor, $N_{g}$ is the installation errors, $n_{\omega }$ is the gyro noise, $\tilde{f}$ is the actual accelerometer outputs, f is the true specific force, b is the accelerometer bias, $S_{a}$ is the scale factor, $N_{a}$ is the installation errors, and $n_{f}$ is the gyro noise.

The gyro and accelerometer biases normally contain two parts, namely, deterministic part and random part. The deterministic part is constant but it differs each time when the sensor is turned on, known as turn on bias in some literatures. The random part varies with time and it is quantified by the bias instability [4,13,22]. The high-end IMU features very small gyro bias instability, e.g., 0.01°/h or even lower, but for MEMS IMU it is very significant, e.g., 10°/h–100°/h or even higher.

The scale factors of gyro and accelerometer cause sensor errors from the true value. Usually the scale factors for gyros and accelerometers are represented by the diagonal matrices in Equations (5) and (6), respectively, and they are quantified in unit of part per million (ppm). Normally the scale factor for MEMS IMU varies as a function of surrounding environments (such as temperature) [21,23].

(5) $$S_{g}=\left[\begin{array}{ccc} K_{gx} & 0 & 0\\ 0 & K_{gy} & 0\\ 0 & 0 & K_{gz} \end{array}\right]$$

(6) $$S_{a}=\left[\begin{array}{ccc} K_{ax} & 0 & 0\\ 0 & K_{ay} & 0\\ 0 & 0 & K_{az} \end{array}\right]$$

where $K_{gx},K_{gy},K_{gz}$ are the scale factors for gyros along X-, Y- and Z-axes, respectively, and $K_{ax},K_{ay},K_{az}$ are the scale factors for accelerometers along X-, Y- and Z-axes, respectively.

Due to the imperfection of inertial sensor assembling and installation, the three sensitive axes of the sensor triad are not perfectly orthogonal with each other, which cause the sensed inertial value of one axis to project into the two other axes. The installation error for gyros and accelerometers can be described by Equations (7) and (8), respectively, and they are quantified using the unit of angle. Similarly, the installation errors are also temperature-dependent [21].

(7) $$N_{g}=\left[\begin{array}{ccc} 0 & K_{gxy} & K_{gxz}\\ K_{gyx} & 0 & K_{gyz}\\ K_{gzx} & K_{gzy} & 0 \end{array}\right]$$

(8) $$N_{a}=\left[\begin{array}{ccc} 0 & K_{axy} & K_{axz}\\ K_{ayx} & 0 & K_{ayz}\\ K_{azx} & K_{azy} & 0 \end{array}\right]$$

where $K_{gij}$ is the installation error between i-axis and j-axis, and $K_{aij}$ is the accelerometer installation error between *i*-axis and *j*-axis (*i*, *j* = *x*, *y*, *z*).

Although the sensor biases, scale factors and installation errors vary in nature, they are considered to be constants in a short time period under stable temperature, e.g., during a complete rotation cycle, to simplify the analysis.

#### 2.2.2. Error Mitigation by Rotating IMU about X Axis

As the IMU rotates about its X-axis with a rate of ω, the transformation matrix between the body frame and the sensor frame can be described by Equation (9).

(9) $$C_{b}^{s}=\left[\begin{array}{ccc} 1 & 0 & 0\\ 0 & \cos\omega t & \sin\omega t\\ 0 & -\sin\omega t & \cos\omega t \end{array}\right]={\left(C_{s}^{b}\right)}^{T}$$

By applying the above transformation matrix, the gyro and accelerometer biases in the navigation frame can be described by Equations (10) and (11), respectively [24,25]. These errors in north and vertical directions are modulated into periodic signals, and the attitude and velocity errors caused by such errors are self-eliminated after a complete rotation cycle as shown in Equations (12) and (13). As the gyro and accelerometer bias in the rotation axis cannot be modulated, the attitude and velocity error in the east direction propagates in the same way as in the conventional INS.

(10) $$d^{n}=C_{b}^{n}C_{s}^{b}d^{s}=\left[\begin{array}{c} d_{x}^{s}\\ d_{y}^{s}\cos\omega t+d_{z}^{s}\sin\omega t\\ -d_{y}^{s}\sin\omega t+d_{z}^{s}\cos\omega t \end{array}\right]$$

(11) $$b^{n}=C_{b}^{n}C_{s}^{b}b^{s}=\left[\begin{array}{c} b_{x}^{s}\\ b_{y}^{s}\cos\omega t+b_{z}^{s}\sin\omega t\\ -b_{y}^{s}\sin\omega t+b_{z}^{s}\cos\omega t \end{array}\right]$$

(12) $$\int_{0}^{T}d^{n}dt=\left[\begin{array}{c} Td_{x}^{s}\\ 0\\ 0 \end{array}\right]$$

(13) $$\int_{0}^{T}b^{n}dt=\left[\begin{array}{c} Tb_{x}^{s}\\ 0\\ 0 \end{array}\right]$$

where $d^{s}={\left[\begin{array}{ccc} d_{x}^{s} & d_{y}^{s} & d_{z}^{s} \end{array}\right]}^{T}$ and $b^{s}={\left[\begin{array}{ccc} b_{x}^{s} & b_{y}^{s} & b_{z}^{s} \end{array}\right]}^{T}$ are the gyro drift and accelerometer bias in IMU frame, respectively; $d^{n}={\left[\begin{array}{ccc} d_{E} & d_{N} & d_{U} \end{array}\right]}^{T}$ and $b^{n}={\left[\begin{array}{ccc} b_{E} & b_{N} & b_{U} \end{array}\right]}^{T}$ are the gyro drift and accelerometer bias in navigation frame, respectively; and $C_{b}^{n}$ is the transformation matrix from body frame to navigation frame.

Although IMU rotation can modulate the sensor constant biases, it also induces additional gyro biases proportional to the rotation rate due to the gyro scale factor of the X-axis as shown in Equation (14). After a complete rotation cycle, the rotation-induced gyro bias results in the accumulated attitude errors as shown in Equation (15). Similarly, the IMU rotation rate will be projected to the Y- and Z-axes due to gyro installation errors, and leads to additional gyro biases as shown in Equation (16). Although the rotation-induced gyro biases can be modulated, and the resulted attitude errors are removed after a complete rotation cycle as shown in Equation (17), it still degrades the navigation solutions within the rotation cycle [17,26].

(14) $$\delta \omega_{SF}^{n}=C_{s}^{n}S_{g}\omega_{is}^{s}=\left[\begin{array}{c} K_{gx}\omega \\ K_{gy}(\omega_{ie}\cos\varphi \frac{1+\cos2\omega t}{2}+\omega_{ie}\sin\varphi \frac{\sin2\omega t}{2})\\ -K_{gz}(-\omega_{ie}\cos\varphi \frac{1-\cos2\omega t}{2}+\omega_{ie}\sin\varphi \frac{\sin2\omega t}{2})\\ K_{gy}(\omega_{ie}\cos\varphi \frac{\sin2\omega t}{2}+\omega_{ie}\sin\varphi \frac{1-\cos2\omega t}{2})\\ +K_{gz}(-\omega_{ie}\cos\varphi \frac{\sin2\omega t}{2}+\omega_{ie}\sin\varphi \frac{1+\cos2\omega t}{2}) \end{array}\right]$$

(15) $$\int_{0}^{T}\delta \omega_{SF}^{n}dt=\left[\begin{array}{c} TK_{gx}\omega \\ \frac{1}{2}T(K_{gy}+K_{gz})\omega_{ie}\cos\varphi \\ \frac{1}{2}T(K_{gy}+K_{gz})\omega_{ie}\sin\varphi \end{array}\right]$$

(16) $$\delta \omega_{N}^{n}=C_{s}^{n}N_{g}\omega_{is}^{s}=\left[\begin{array}{c} \;\begin{array}{l} K_{gxy}(\omega_{ie}\cos\varphi \cos\omega t+\omega_{ie}\sin\varphi \sin\omega t)+K_{gxz}(-\omega_{ie}\cos\varphi \sin\omega t\\ +\omega_{ie}\sin\varphi \cos\omega t) \end{array}\\ \begin{array}{l} K_{gyx}\omega \cos\omega t-K_{gzx}\omega \sin\omega t+K_{gyz}\omega_{ie}(\sin\varphi \frac{1+\cos2\omega t}{2}-\cos\varphi \frac{\sin2\omega t}{2})\\ -K_{gzy}\omega_{ie}(\cos\varphi \frac{\sin2\omega t}{2}+\sin\varphi \frac{1-\cos2\omega t}{2}) \end{array}\\ \begin{array}{l} K_{gyx}\omega \sin\omega t+K_{gzx}\omega \cos\omega t+K_{gyz}\omega_{ie}(\sin\varphi \frac{\sin2\omega t}{2}-\cos\varphi \frac{1-\cos2\omega t}{2})\\ +K_{gzy}\omega_{ie}(\cos\varphi \frac{1+\cos2\omega t}{2}+\sin\varphi \frac{\sin2\omega t}{2}) \end{array} \end{array}\right]$$

(17) $$\int_{0}^{T}\delta \omega_{N}^{n}=\left[\begin{array}{c} 0\\ \frac{1}{2}(K_{gyz}-K_{gzy})\omega_{ie}\sin\varphi \\ \frac{1}{2}(-K_{gyz}+K_{gzy})\omega_{ie}\cos\varphi \end{array}\right]$$

where $\delta \omega_{SF}^{n}$ is the sensor error caused by the scale factor, $\omega_{ie}$ is the earth rotation rate in the navigation frame, and $\delta \omega_{N}^{n}$ is the gyro error caused by the installation error.

#### 2.2.3. Error Mitigation by Rotating IMU about Y and Z Axis

When IMU rotates about the Y-axis, both gyro and accelerometer biases in X- and Z-axes are modulated, so the attitude and velocity errors caused by those errors are automatically removed in east and vertical directions after a complete rotation cycle, while the errors in north direction propagate in the same way as in conventional INS, as shown in Equations (18) and (19). Similarly, the IMU rotation also induces the errors in the Y-axis due to the scale factor of the rotation axis, and the errors in the X- and Z-axes due to the gyro installation errors, as shown in Equations (20) and (21), respectively [26].

(18) $$d^{n}=C_{b}^{n}C_{s}^{b}d^{s}=\left[\begin{array}{c} d_{x}^{s}\cos\omega t-d_{z}^{s}\sin\omega t\\ d_{y}^{s}\\ d_{x}^{s}\sin\omega t+d_{z}^{s}\cos\omega t \end{array}\right]$$

(19) $$b^{n}=C_{b}^{n}C_{s}^{b}b^{s}=\left[\begin{array}{c} b_{x}^{s}\cos\omega t-b_{z}^{s}\sin\omega t\\ b_{y}^{s}\\ b_{x}^{s}\sin\omega t+b_{z}^{s}\cos\omega t \end{array}\right]$$

(20) $$\delta \omega_{SF}^{n}=\left[\begin{array}{c} -K_{gx}\omega_{ie}\sin\varphi \frac{\sin2\omega t}{2}+K_{gz}\omega_{ie}\sin\varphi \frac{\sin2\omega t}{2}\\ K_{gy}(\omega_{ie}\cos\varphi +\omega )\\ K_{gx}\omega_{ie}\sin\varphi \frac{1-\cos2\omega t}{2}+K_{gz}\omega_{ie}\sin\varphi \frac{1+\cos2\omega t}{2} \end{array}\right]$$

(21) $$\delta \omega_{N}^{n}=\left[\begin{array}{c} \begin{array}{l} K_{gxy}(\omega_{ie}\cos\varphi +\omega )\cos\omega t+K_{gxz}\omega_{ie}\sin\varphi \cos^{2}\omega t-K_{gzx}\omega_{ie}\sin\varphi \sin^{2}\omega t\\ +K_{gzy}(\omega_{ie}\cos\varphi +\omega )\sin\omega t \end{array}\\ \begin{array}{c} -K_{gyx}\omega_{ie}\sin\varphi \sin\omega t+K_{gyz}\omega_{ie}\sin\varphi \cos\omega t \end{array}\\ \begin{array}{l} -K_{gxy}(\omega_{ie}\cos\varphi +\omega )\sin\omega t-K_{gxz}\omega_{ie}\sin\varphi \frac{\sin2\omega t}{2}-K_{gzx}\omega_{ie}\sin\varphi \frac{\sin2\omega t}{2}\\ +K_{gzy}(\omega_{ie}\cos\varphi +\omega )\cos\omega t \end{array} \end{array}\right]$$

The gyro and accelerometer biases in X- and Y-axes are modulated when IMU rotates about Z-axis, therefore the attitude and velocity errors caused by such errors are self-eliminated in the east-north plane after a complete rotation cycle, though the errors in vertical direction still propagate in the same way as in conventional INS, as shown in Equations (22) and (23). Because of the gyro scale factor in the Z-axis and the installation errors, the IMU rotation also induces gyro biases, which can be described by Equations (24) and (25) [17,26].

(22) $$d^{n}=C_{b}^{n}C_{s}^{b}d^{s}=\left[\begin{array}{c} d_{x}^{s}\cos\omega t+d_{y}^{s}\sin\omega t\\ d_{x}^{s}\sin\omega t-d_{y}^{s}\cos\omega t\\ d_{z}^{s} \end{array}\right]$$

(23) $$b^{n}=C_{b}^{n}C_{s}^{b}b^{s}=\left[\begin{array}{c} b_{x}^{s}\cos\omega t+b_{y}^{s}\sin\omega t\\ b_{x}^{s}\sin\omega t-b_{y}^{s}\cos\omega t\\ b_{z}^{s} \end{array}\right]$$

(24) $$\delta \omega_{SF}^{n}=\left[\begin{array}{c} (K_{gx}-K_{gy})\omega_{ie}\cos\varphi \sin\omega t\cos\omega t\\ (K_{gx}\sin^{2}\omega t+K_{gy}\cos^{2}\omega t)\omega_{ie}\cos\varphi \\ K_{gz}(\omega_{ie}\sin\varphi +\omega ) \end{array}\right]$$

(25) $$\delta \omega_{N}^{n}=\left[\begin{array}{c} \omega_{ie}\cos\varphi (K_{gxy}\cos^{2}\omega t-K_{gyx}\sin^{2}\omega t)+(\omega_{ie}\sin\varphi +\omega )(K_{gxz}\cos\omega t-K_{gyz}\sin\omega t)\\ \omega_{ie}\cos\varphi \sin2\omega t(K_{gxy}+K_{gyx})/2+(\omega_{ie}\sin\varphi +\omega )(K_{gxz}\sin\omega t+K_{gyz}\cos\omega t)\\ K_{gzx}\omega_{ie}\cos\varphi \sin\omega t+K_{gzy}\omega_{ie}\cos\varphi \cos\omega t \end{array}\right]$$

Table 1 summarizes the velocity errors caused by accelerometer biases and the attitude errors caused by gyro biases, gyro scale factors and gyro installation errors after a complete rotation cycle for IMU rotations about the X-, Y- and Z-axes.

For the IMU rotation about the X-axis, the unmodulated accelerometer bias causes the linear velocity errors and quadratic position errors over time in the east direction, as shown in Equation (26). The unmodulated gyro bias in the X-axis results in accumulated attitude error in the east direction, which causes projection error of the local gravity on the north direction. Eventually, it leads to quadratic velocity errors and cubic position errors over time as shown in Equation (27) [3,6]. Although the attitude errors caused by gyro scale factors can be found in the east, north and vertical directions, the east component is much more significant than other two components, as the IMU rotation rate is usually much greater than the earth rotation rate. Similar to the gyro biases, the rotation-induced gyro errors (due to the scale factor) in the east direction also leads to the quadratic velocity errors and cubic position errors in the north direction. As the rotation-induced gyro errors in the Y- and Z-axes due to the installation errors are modulated after a complete rotation cycle, the effect of attitude errors on the velocity and position errors are limited.

Similar to the IMU rotation about the X-axis, the unmodulated accelerometer bias in the Y-axis leads to the linear velocity errors and quadratic position errors in the north direction, while the unmodulated gyro bias and scale factor in the rotation axis result in the quadratic velocity errors and cubic position errors in the east direction, for the IMU rotation about the Y-axis.

For the IMU rotation about the Z-axis, the unmodulated accelerometer bias causes the linear velocity and quadratic position errors in the vertical direction, while the unmodulated gyro bias and scale factor of the rotation axis results in the azimuth errors, which lead to the horizontal velocity and position errors. For the IMU rotation about the X or Y-axis, as the velocity and position errors in the east or north directions accumulate quadratically and cubically over time, they are mostly much greater than the ones for the IMU rotation about the Z-axis, which is related to the coupling of the azimuth errors and the vehicle dynamics (usually can be expressed by a sine function times the vehicle velocity or displacement). Therefore, the IMU rotation about the Z-axis is more suitable than other two rotation schemes for navigation applications. The rest of the paper will concentrate on the use of this rotation scheme.

(26) $$\delta V_{E}=\int b_{x}^{s}dt=b_{x}^{s}t,\delta P_{E}=\int \delta V_{E}dt=\int b_{x}^{s}tdt=\frac{1}{2}b_{x}^{s}t^{2}$$

(27) $$\delta V_{N}=\int g\varepsilon_{E}dt=\int gd_{x}^{s}tdt=\frac{1}{2}gd_{x}^{s}t^{2},\delta P_{N}=\int \delta V_{N}dt=\int \frac{1}{2}gd_{x}^{s}t^{2}dt=\frac{1}{6}gd_{x}^{s}t^{3}$$

Other conclusions are summarized as follows:

(1) The IMU rotation modulates the constant biases of inertial sensors that perpendicular to the rotation axis, and the attitude and velocity errors caused by such biases are self-eliminated after a complete rotation cycle;
(2) The constant biases of inertial sensors in the rotation axis cannot be modulated, and the attitude and velocity errors caused by such errors propagate in the same way as in conventional INS;
(3) The IMU rotation induces an extra error in the gyro of the rotation axis due to gyro scale factor, and this error results in accumulated attitude errors in the direction of the corresponding rotation axis;
(4) The IMU rotation also induces extra errors in the gyros that are perpendicular to the rotation axis due to gyro installation errors, resulting in attitude and velocity errors.
(5) As the rotation-induced errors are proportional to the rotation rate, which is usually much more significant than earth rotation rate, a calibration process is required to remove the rotation induced errors.

## 3. Calibration for the MEMS IMU

As the IMU rotation induces additional gyro biases, which eventually leads to navigation errors [17,26], a calibration process is proposed for rotary INS with IMU rotation about Z-axis. Although the full gyro scale factor and installation errors are represented by 9 parameters according to Equations (5) and (6), the proposed method only calibrate the gyro scale factor of Z-axis, $K_{gz}$, and the installation errors, $K_{gxz},K_{gyz}$, which motivate significant gyro errors when IMU rotates about Z-axis.

### 3.1. Gyro Error Model for Calibration

As the IMU rotation does not introduce any linear motion of IMU, the theoretical gyro outputs in body frame can be described by Equation (28), when body frame is static relative to local level frame.

(28) $$\omega_{ib}^{b}=C_{n}^{b}\omega_{ib}^{n}=C_{n}^{b}\omega_{ie}^{n}=C_{n}^{b}\left[\begin{array}{c} 0\\ \omega_{ie}\cos\varphi \\ \omega_{ie}\sin\varphi \end{array}\right]=\left[\begin{array}{c} p\omega_{ie}\\ q\omega_{ie}\\ r\omega_{ie} \end{array}\right]$$

where and they satisfy the equality equation $p^{2}+q^{2}+r^{2}=1$.

The theoretical and actual gyro outputs in the sensor frame can be described by Equations (29) and (30), respectively, and the gyro errors are given in Equation (31).

(29) $$\omega_{is}^{s}=C_{b}^{s}\omega_{ib}^{b}+\omega_{bs}^{s}=\left[\begin{array}{c} \omega_{ie}(p\cos\omega t+q\sin\omega t)\\ \omega_{ie}(-p\sin\omega t+q\cos\omega t)\\ r\omega_{ie}+\omega \end{array}\right]$$

(30) $${\tilde{\omega }}_{is}^{s}=\omega_{is}^{s}+N\omega_{is}^{s}+d^{s}$$

(31) $$\delta \omega_{is}^{s}=\left[\begin{array}{c} S_{gx}\omega_{ie}(p\cos\omega t+q\sin\omega t)+K_{gxy}\omega_{ie}(-p\sin\omega t+q\cos\omega t)+K_{gxz}(r\omega_{ie}+\omega )+d_{x}^{s}\\ K_{gyx}\omega_{ie}(p\cos\omega t+q\sin\omega t)+S_{gy}\omega_{ie}(-p\sin\omega t+q\cos\omega t)+K_{gyz}(r\omega_{ie}+\omega )+d_{y}^{s}\\ K_{gzx}\omega_{ie}(p\cos\omega t+q\sin\omega t)+K_{gzy}\omega_{ie}(-p\sin\omega t+q\cos\omega t)+S_{gz}(r\omega_{ie}+\omega )+d_{z}^{s} \end{array}\right]$$

where $N=\left[\begin{array}{ccc} S_{gx} & K_{gxy} & K_{gxz}\\ K_{gyx} & S_{gy} & K_{gyz}\\ K_{gzx} & K_{gzy} & S_{gz} \end{array}\right]$ is a 3 × 3 matrix represents the combination of gyro scale factor and installation errors and $d^{s}={\left[\begin{array}{ccc} d_{x}^{s} & d_{y}^{s} & d_{z}^{s} \end{array}\right]}^{T}$ is the gyro biases in sensor frame.

The earth rotation rate can be ignored in the above error model because: (1) the earth rotation rate cannot be sensed in most MEMS IMU due to their significant gyro bias instability and noise; and (2) the IMU rotation rate is much more significant than earth rotation rate. By ignoring the earth rotation rate, the above gyro error model can be simplified as shown in Equation (32).

(32) $$\delta \omega_{is}^{s}=\left[\begin{array}{c} K_{gxz}\omega +d_{x}^{s}\\ K_{gyz}\omega +d_{y}^{s}\\ S_{gz}\omega +d_{z}^{s} \end{array}\right]$$

With different IMU rotation rates, the least square algorithm is applied to estimate the gyro scale factor, $K_{gz}$, the gyro installation errors, $K_{gxz}$, $K_{gyz}$, as well as the gyro biases, ${\left[\begin{array}{ccc} d_{x}^{s} & d_{y}^{s} & d_{z}^{s} \end{array}\right]}^{T}$, based on the error model given in Equation (32). The measurements are the gyro readings which can be described by Equation (33), and the measurement model for a single measurement is given by Equation (34).

(33) $$Z_{j}={\tilde{\omega }}_{is}^{s}-\omega_{is}^{s}\approx \left[\begin{array}{c} {\tilde{\omega }}_{is,x}^{s}\\ {\tilde{\omega }}_{is,y}^{s}\\ {\tilde{\omega }}_{is,z}^{s}-\omega_{j} \end{array}\right]$$

(34) $$Z_{j}=H_{j}X=\left[\begin{array}{cccccc} \omega_{j} & 0 & 0 & 1 & 0 & 0\\ 0 & \omega_{j} & 0 & 0 & 1 & 0\\ 0 & 0 & \omega_{j} & 0 & 0 & 1 \end{array}\right]\left[\begin{array}{c} K_{gxz}\\ K_{gyz}\\ S_{gz}\\ d_{x}^{s}\\ d_{y}^{s}\\ d_{z}^{s} \end{array}\right]$$

where $Z_{j}$ represent the jth measurement, and $\omega_{j}$ represents the IMU rotation rate with respect to the $Z_{j}$.

### 3.2. Implementation of Calibration Process

The calibration process can be described as two steps: (1) the tested IMU remains still on the rotation platform for 60 s; and (2) the IMU rotates along with the rotation platform at a designated rotation rate about Z-axis for 60 s. The total calibration time is 2 min. According to the error model in Equation (32), the gyro biases can be estimated from the inertial data collected in static period, while the gyro scale factor, $K_{gz}$, and installation errors, $K_{gxz},K_{gyz}$, can be estimated from the collected data in rotation period. It is worthy to mention that, the MEMS IMU usually features significant non-linearity errors [27,28], so the designated rotation rate in calibration process should be the same as the one employed in rotation scheme. For example, for the MEMS-based rotary INS with IMU rotation about Z-axis at the rate of 10°/s, the calibration process should employ the same rotation rate to estimate the gyro scale factor and installation errors.

The error sources for the calibration error include the gyro sensor noise, the gyro bias instability (gyro biases are not constants but time-correlated variables), and the ignored earth rotation rate. The calibration errors of gyro biases caused by noise can be calculated using Equation (35), and the calibration errors of scale factor and installation errors caused by noise are given by Equation (36) [21]. Apparently, the longer calibration time can reduce the calibration errors, however it may increase the effect of gyro bias instability on calibration results. The reason we choose the 60 s for calibration time in each step will be further discussed in Section 4 along with the noise level and gyro bias instability of tested IMU.

(35) $$\sigma_{b}=ARW/\sqrt{T_{static}}$$

(36) $$\sigma_{s}=ARW\cdot \sqrt{T_{rotation}}/\alpha_{rotation}$$

where *ARW* is the angular random walk of gyros, $T_{static}$ is the time period that IMU remains still, $T_{rotation}$ is the time period that IMU rotation, and $\alpha_{rotation}$ is the rotated angle of IMU.

It should be noted that the calibration method above is proposed to estimate gyro errors before each use of the rotary system. It requires the vehicle remains still during the calibration process, and this limits its use for on-line calibration of the rotary system. For on-line calibration, the external measurements are required, and an extended Kalman filter (EKF) is usually employed to estimate the sensor errors.

## 4. Testing MEMS IMU on a Tri-Axial Rotation Table

**Figure 3:**
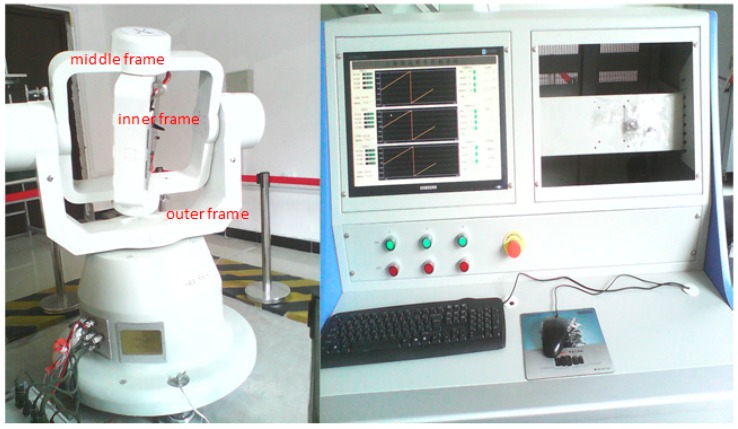
Tri-axial rotation platform.

By using a tri-axial rotation table as the rotation platform, two MEMS IMUs, namely MTi-G and NAV440 are tested. The rotation platform consists of a tri-axial rotation table and a console (computer) as shown in Figure 3. The tri-axial rotation table has three rotational frames, namely, outer frame, middle frame and inner frame. The console controls the position and rotation of these frames.

**Figure 4:**
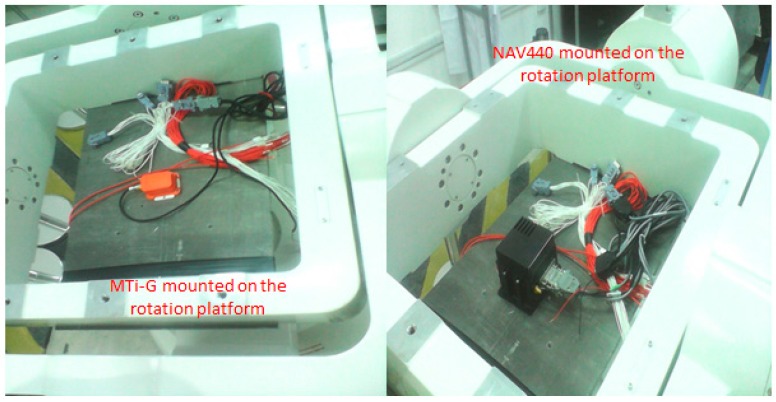
Installation of Micro-Electron-Mechanical System (MEMS) IMU on tri-axial rotation table.

**Figure 5:**
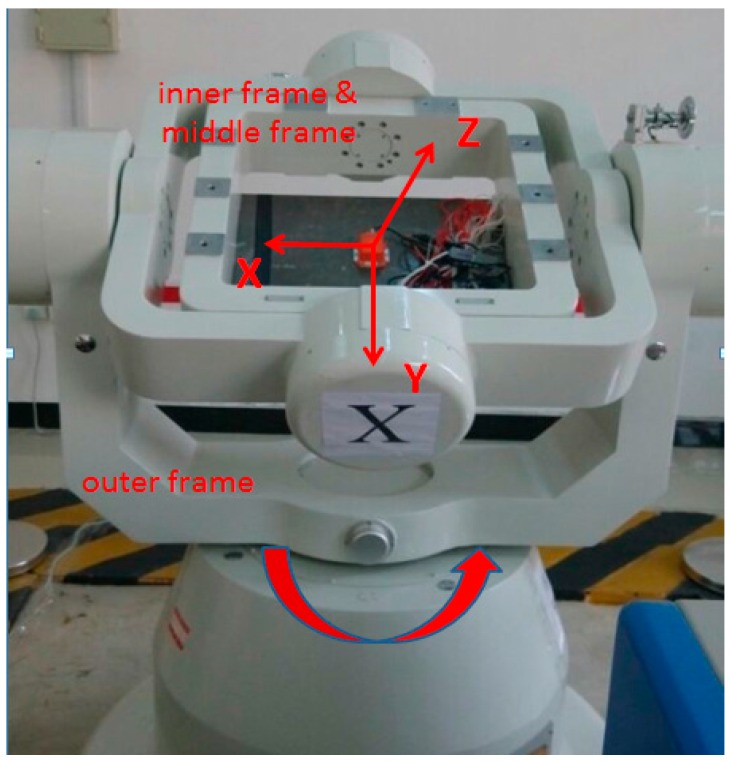
Rotation table set-up for IMU rotation about Z-axis.

The MEMS IMUs are firmly installed on a piece of metal underneath the inner frame by screws as shown in Figure 4. Different rotation schemes, such as rotation about X-, Y- and Z-axes can be implemented by rotating the three frames. For example, with the IMU axis defined as shown in Figure 5, the rotation of outer frame rotates the IMU about its Z-axis when both the middle and inner frames remain at the level position (or angle position of 0°). An initialization process, after which both middle and inner frames are in the level position and the rotation axis of inner frame points to north direction, is required for the rotation table. Apparently, with IMU installations as shown in Figure 5, the IMU body frame is aligned to local level frame after the initialization process.

Table 2 and Table 3 summarize the technical parameters of the rotation platform and the characteristics of the tested IMU, respectively. All data are provided from the manufactures and available on their official website. Although the gyro white noise of NAV440 is not provided, the Allan variance method was utilized to obtain this value, which is about 0.4°/$\sqrt{h}$ [13].

The frame rotation angle data (IMU rotation angle between body frame and sensor frame) and inertial data are recorded by the console with data rates of 50 Hz and 100 Hz, respectively. The frame rotation angle will be used to calculate transformation matrix between body frame and sensor frame, which are necessary to solve the attitude solutions.

**Figure 6:**
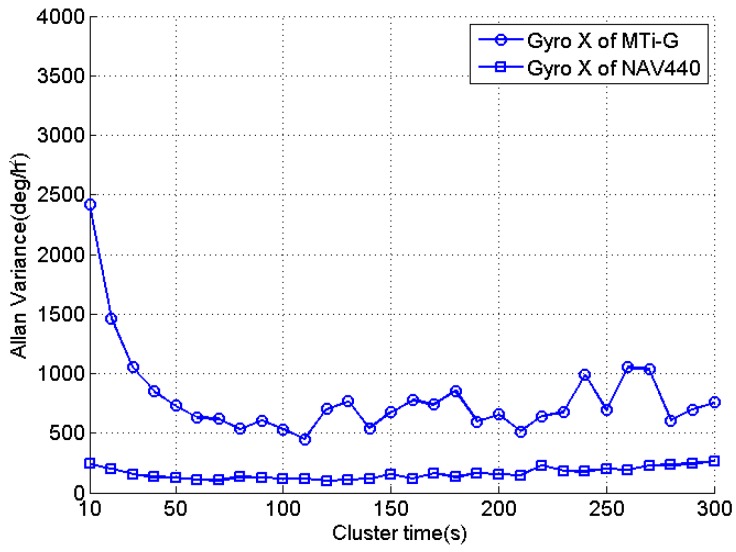
Allan variances of gyro data in X-axis for MTi-G and NAV440.

The Allan variance method was also employed to study the random variations of the sensor biases as shown in Figure 6, which presents the calculated Allan variance of gyro data in the X-axis for both tested IMUs. The cluster time (or averaging time) ranges from 10 s to 300 s, and the most significant bias variance was observed at the cluster time of 10 s for MTi-G due to its high noise level. As the cluster time increases, the bias variances are reduced as the noises are eliminated by the averaging process. For both IMUs, the bias variances reach their minimums at the cluster time of around 120 s (about 24°/h and 11°/h for MTi-G and NAV440, respectively). For the longer cluster time, the bias variances are fluctuated due to the rate random walk, the exponentially correlated noise and the sinusoidal noise [29]. For the gyro data in the Y- and Z-axes, similar results are obtained.

The conventional INS static tests and the rotary INS static tests are conducted using both MEMS IMUs in a laboratory. The conventional INS static tests, in which the inertial data are collected while the MEMS IMU remains still on the rotation table for 6 min (the data from the 1st minute will be used to derive the gyro bias estimates), are conducted to investigate how fast the inertial errors accumulate with time in a non-rotary system and to provide a comparison to the rotary INS. The rotary INS static tests are conducted to study the feasibility of the MEMS-based rotary system, and to verify the inertial error mitigation performance through IMU rotations. Although higher IMU rotation rates can more effectively mitigate the navigation errors in theory [24,25], the effect of IMU rotation rate on navigation errors needs to be investigated for a MEMS-based rotary system, as the MEMS IMU features significant bias instability and noise level. Ten individual tests are conducted for the rotary INS static tests. In each individual test, the IMU rotates about Z-axis at a designated rate with a calibration process conducted in the beginning. The designated rotation rates for the ten individual tests are 10°/s, 20°/s, 30°/s, 40°/s, 50°/s, 60°/s, 70°/s, 80°/s, 90°/s and 100°/s, respectively. The time length of the rotated data for each individual test is 5 min (not including the calibration time), as after that the position errors are already accumulated to hundreds or thousands of meters. As mentioned in Section 3, the IMU rotation rate employed in the calibration process is the same as the one in the corresponding individual test.

### 4.1. Results of Conventional INS Static Tests

The means of the gyro data in three axes collected in the 1st minute provide the estimates of the gyro biases [30]. Based on the initial position, velocity and attitude from external information (body frame is aligned to local level frame after the initialization of rotation table), the navigation solutions are calculated based on the collected inertial data. As expected, the position, velocity and attitude errors accumulate fast with time. The Root mean square (RMS) values of the navigation errors for MTi-G and NAV440 are summarized in Table 4 and Table 5, respectively. After 5 min, the horizontal position errors accumulate to several kilometers for both MEMS IMUs.

### 4.2. Results of Rotary INS Static Tests

In each individual test, the gyro scale factor of Z-axis, $K_{gz}$, the installation errors, $K_{gxz},K_{gyz}$, and the gyro biases are estimated for MTi-G and NAV440 in the calibration process. Based on the gyro noise level of tested IMUs given in Table 3, the calibration errors caused by gyro noise for both IMUs are calculated and given in Table 6. It should be noted that the unit of installation errors are converted to ppm, and the calibration errors of scale factor and installation errors can also be converted to degree per hour. With the time length of 60 s, the calibration errors of gyro biases caused by the noises are 23°/h and 3°/h for MTi-G and NAV440, respectively, and similar calibration errors are also obtained for scale factor and installation errors.

The major calibration error sources contain the gyro noises and the gyro bias random variations. Although longer calibration time reduces calibration errors caused by the noise, it also increases the effect of gyro bias variations on calibration results. Therefore, the choosing of calibration time is a compromise of the two factors. For both tested IMUs, the calibration time is chosen as 120 s (60 s in each step) and the reason is that the calibration errors caused by the gyro noise are comparable to the minimum bias variations for the cluster time of 120 s as shown in Figure 6.

Table 7 summarizes the mean of the gyro data in both IMUs after the calibration process for each individual test. As we can see, the mean values of the gyro data in the X- and Y-axes are close to zero and the means of the gyro data in the Z-axis are close to the rotation rate after the calibration, which prove the rotation induced gyro biases and turn on biases are removed.

The rotary INS results are derived with two different data processing strategies, namely, partially calibrated data processing and fully calibrated data processing, to verify the effect of gyro scale factor and installation errors on navigation errors. For the partially calibrated data processing, the rotary navigation solutions are derived with only the estimates of gyro biases from the calibration results, so the gyro scale factor and installation errors remain in the rotary inertial data. For the fully calibrated data processing, the estimates of gyro installation errors, scale factor and biases are applied to derive the rotary INS solutions.

#### 4.2.1. MTi-G Results

**Figure 7:**
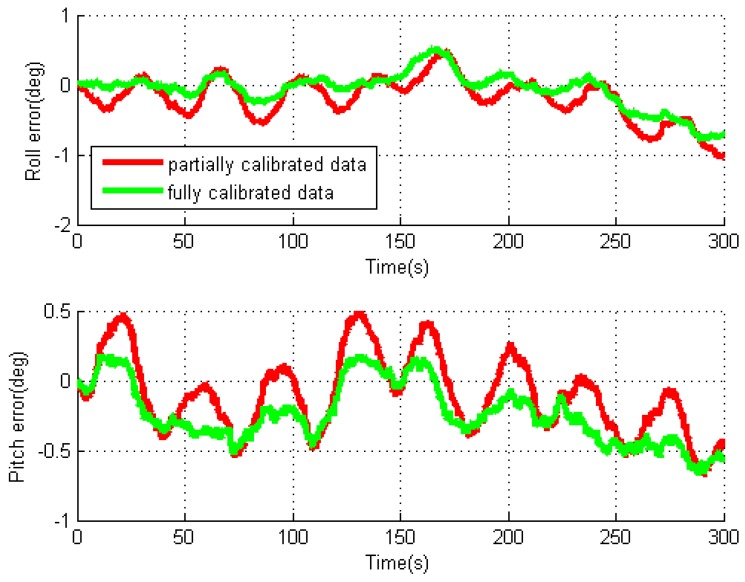
Roll and pitch errors for rotary INS with MTi-G.

By using two data processing methods, the obtained roll and pitch errors for the individual test with rotation rate of 10°/s are given in Figure 7. For the partially calibrated data processing, the attitude errors are modulated into oscillating signals because of the modulation of the gyro errors in X- and Y-axes through IMU rotation, and the oscillating period is the same as the rotation period. As the gyro biases are already removed, the remaining errors mostly consist of bias residuals and rotation-induced errors due to gyro installation errors. Similar characteristics can be observed in the horizontal velocity errors. For the fully calibrated data processing, the oscillating amplitudes are greatly reduced as shown in the figure. This is because the removal of gyro installation errors eliminates the effect of rotation-induced errors on navigation solutions.

**Figure 8:**
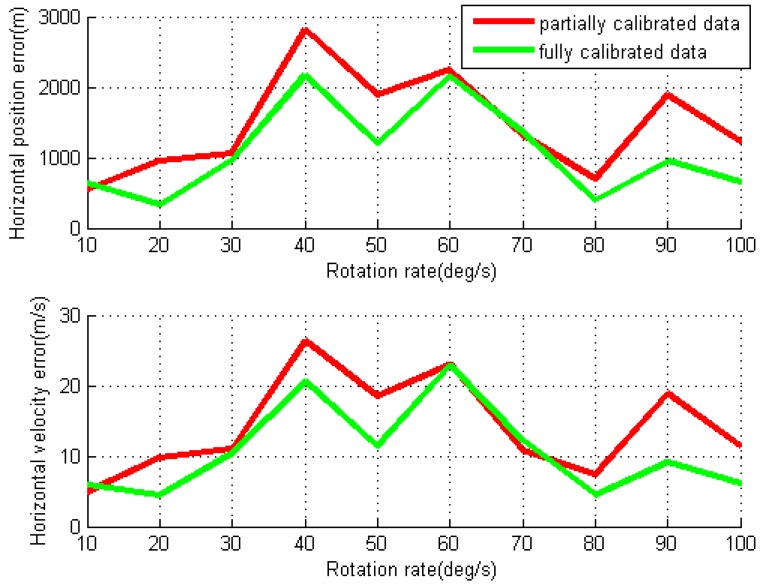
Root mean square (RMS) values of horizontal position and velocity errors for rotary INS with different IMU rotation rates using MTi-G.

**Figure 9:**
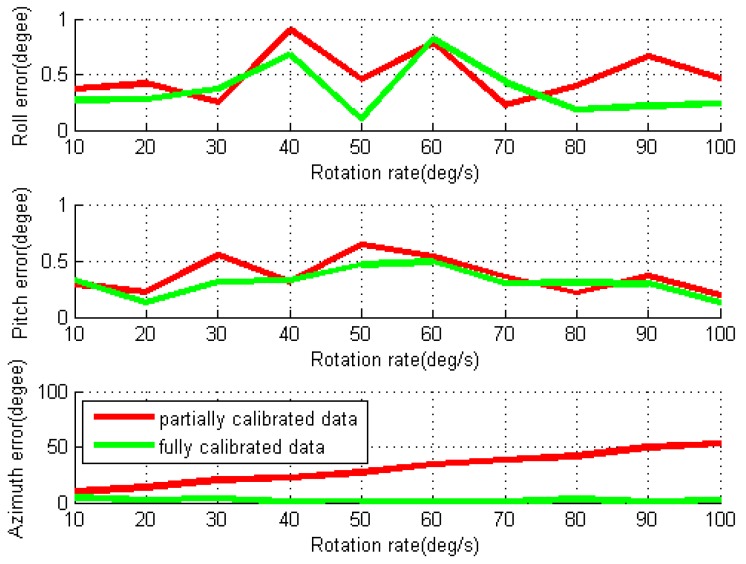
RMS values of attitude errors for rotary INS with different IMU rotation rates using MTi-G.

For each individual test, the RMS values of the position, velocity and attitude errors are calculated using partially and fully data processing strategies, as shown in Figure 8 and Figure 9. Comparing to the errors in the conventional INS tests, the horizontal position, velocity, roll and pitch errors are reduced by modulation accelerometer and gyro errors in X- and Y-axes through IMU rotation in the partially calibrated case, although the gyro scale factor and installation errors are not removed yet. The major error sources of the navigation errors are gyro bias residuals (as the gyro biases vary with time) and the induced gyro biases by IMU rotation. As shown in Figure 9, the IMU rotation induces the gyro bias in Z-axis, which leads to azimuth error almost linearly increases over the rotation rate. The mean values of the RMS errors for horizontal position and velocity for all tested rotation rates are 1525.8 m and 14.6 m/s, respectively.

For the fully calibrated data processing, the removal of the rotation-induced gyro errors in the X- and Y-axes further reduce the roll and pitch errors, as well as the horizontal position and velocity errors, comparing to the results in the partially calibrated case. The azimuth errors are also significantly reduced because of the estimation of the gyro scale factor in the Z-axis. The mean value of RMS for the horizontal position errors and the velocity errors for all tests are further reduced to 1057.2 m and 10.4 m/s, respectively. The roll and pitch errors are found much smaller than the azimuth errors. This is because the gyro errors in the X- and Y-axes are modulated by IMU rotation about the Z-axis, while such errors in the Z-axis cannot be modulated. Although a higher IMU rotation rate can more effectively modulate sensor biases and mitigate error accumulations for FOG or RLG-based system [24,25], it can be noted that the navigation errors are not strongly related to the IMU rotation rate for MTi-G. This is because the MTi-G is a very low-cost MEMS IMU, which features significant bias instability (e.g., 3600°/h, as shown in Table 3) and high noise level (e.g., 3.0°/$\sqrt{h}$, as shown in Table 3). As a result, they affect the error mitigation performance in rotary system.

#### 4.2.2. Nav440 Results

**Figure 10:**
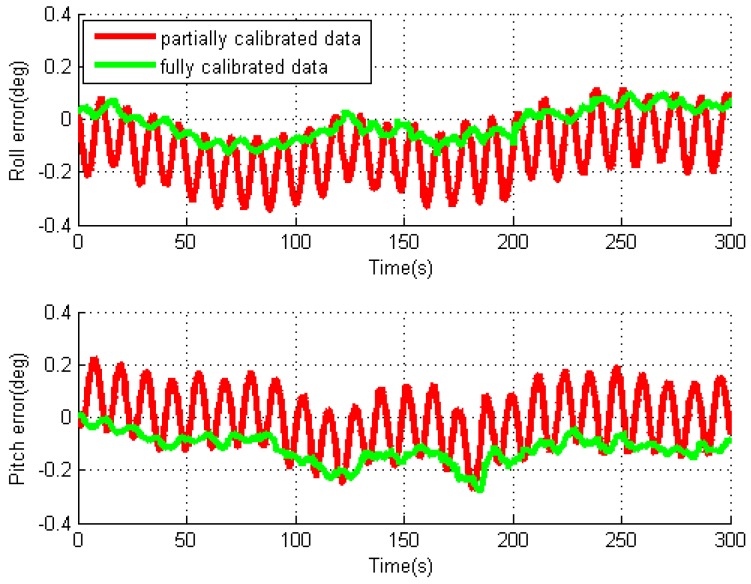
Roll and pitch errors for rotary INS with NAV440.

The rotated Nav440 data are also processed using the two data processing methods, and the obtained roll and pitch errors for the individual test with rotation rate of 30°/s are given in Figure 10. Similarly, the attitude errors are modulated into oscillating signals through IMU rotation, and the oscillating amplitude is greatly reduced in the fully calibrated case. Based on Figure 10, we can see that the greater oscillating amplitude in the partially calibrated case is mainly caused by rotation-induced errors due to gyro installation errors, while the much smaller oscillating amplitude in the fully calibrated case is caused by gyro bias residuals. Similar characteristics can be observed in horizontal velocity errors.

**Figure 11:**
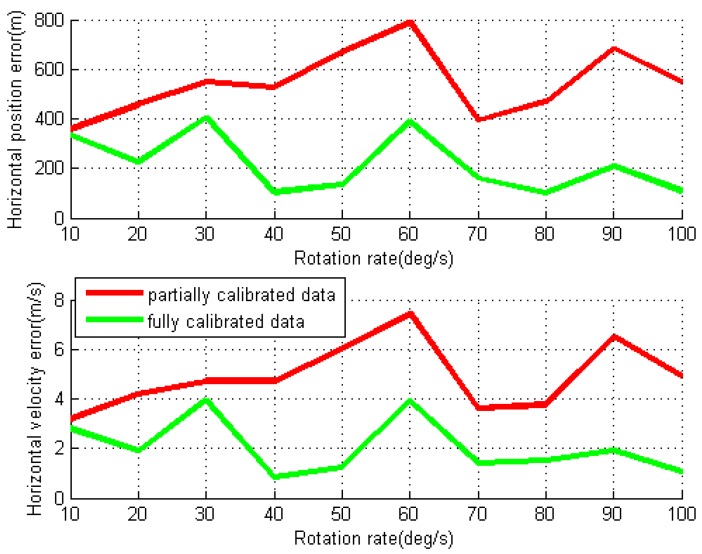
RMS values of horizontal position and velocity errors for rotary INS with different IMU rotation rates using NAV440.

**Figure 12:**
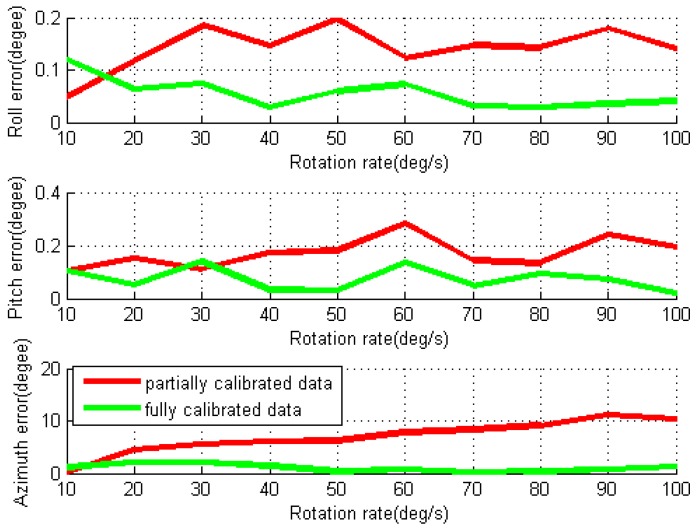
RMS values of attitude errors for rotary INS with different IMU rotation rates using NAV440.

By using two different data processing methods, the obtained RMS values of navigation errors for each individual test are given in Figure 11 and Figure 12. For the partially calibrated data processing, the horizontal position and velocity errors as well as pitch and roll errors are reduced compared to the ones in conventional test, though the azimuth error still grows almost linearly over the IMU rotation rate due to the rotation-induced gyro errors in Z-axis. The mean values of the RMS errors for horizontal position and velocity are 544.8 m and 4.9 m/s, respectively. With the removal of gyro installation errors and scale factors in fully calibrated data processing, all navigation errors are further reduced, and the mean of RMS horizontal position and velocity errors are dropped to 213.8 m and 2.2 m/s, respectively.

It can be noted that the magnitude of the roll and pitch errors are nearly one order smaller than the azimuth errors due to the modulation of gyro errors in the X- and Y-axes. As the NAV440’s gyro bias instability and noise level are much lower than MTi-G, much smaller navigation errors are obtained for the rotary system based on NAV440.

Based on the results from the conventional INS static tests and the rotary INS static tests with two different MEMS IMUs, we can see that IMU rotations can modulate the sensor errors, and mitigate their effect on navigation solutions. The gyro scale factor and installation errors will cause additional navigation errors by IMU rotations, and their calibration is therefore necessary for a MEMS-based rotary system in order to achieve better navigation performance. The rotation rate is not the only factor that affects the error modulation, which will also be affected by the sensor errors, such as the bias instability and the noise. The results also indicate that the sensor errors can be more efficiently modulated for the MEMS IMUs that have smaller bias instability and lower noise level.

## 5. Conclusions

This paper investigates the mitigation of navigation errors due to significant MEMS inertial sensor errors by rotating IMU. Three IMU rotation schemes, each rotating about X-, Y- and Z-axes, respectively, are investigated. The related mathematical equations are derived and analyzed for each rotation scheme. A calibration method is proposed to remove the sensor biases, as well as gyro scale factors and installation errors for a MEMS-based rotary system. The tests with two MEMS IMUs on a tri-axial rotation table are conducted to verify the feasibility of MEMS-based rotary INS. The results indicate that with efficient data processing strategy, the IMU rotation can effectively modulate sensor errors and mitigate their effects on navigation solutions for a low-cost MEMS IMU. Kinematic field tests and other factors, such as vibration and wobble, that may affect the error mitigation in rotary INS, will be investigated in the future research.

## Figures and Tables

**Table 1:** Errors after a complete rotation cycle for IMU rotation about X-, Y- and Z-axes.

Errors After a Complete Rotation Cycle	Rotation About X Axis	Rotation About Y Axis	Rotation About Z Axis
Accelerometer biases	$\left[\begin{array}{c} Tb_{x}^{s}\\ 0\\ 0 \end{array}\right]$	$\left[\begin{array}{c} 0\\ Tb_{y}^{s}\\ 0 \end{array}\right]$	$\left[\begin{array}{c} 0\\ 0\\ Tb_{z}^{s} \end{array}\right]$
Gyro biases	$\left[\begin{array}{c} Td_{x}^{s}\\ 0\\ 0 \end{array}\right]$	$\left[\begin{array}{c} 0\\ Td_{y}^{s}\\ 0 \end{array}\right]$	$\left[\begin{array}{c} 0\\ 0\\ Td_{z}^{s} \end{array}\right]$
Gyro scale factors	$\left[\begin{array}{c} TK_{gx}\omega \\ \frac{1}{2}T(K_{gy}+K_{gz})\omega_{ie}\cos\varphi \\ \frac{1}{2}T(K_{gy}+K_{gz})\omega_{ie}\sin\varphi \end{array}\right]$	$\left[\begin{array}{c} 0\\ TK_{gy}(\omega_{ie}\cos\varphi +\omega )\\ \frac{T}{2}(K_{gx}+K_{gz})\omega_{ie}\sin\varphi \end{array}\right]$	$\left[\begin{array}{c} 0\\ \frac{T}{2}(K_{gx}+K_{gy})\omega_{ie}\cos\varphi \\ K_{gz}(\omega_{ie}\sin\varphi +\omega )T \end{array}\right]$
Gyro installation errors	$\left[\begin{array}{c} 0\\ \frac{1}{2}(K_{gyz}-K_{gzy})\omega_{ie}\sin\varphi \\ \frac{1}{2}(-K_{gyz}+K_{gzy})\omega_{ie}\cos\varphi \end{array}\right]$	$\left[\begin{array}{c} \frac{T}{2}(K_{gxz}-K_{gzx})\omega_{ie}\sin\varphi \\ 0\\ 0 \end{array}\right]$	$\left[\begin{array}{c} \frac{T}{2}(K_{gxy}-K_{gyx})\omega_{ie}\cos\varphi \\ 0\\ 0 \end{array}\right]$

**Table 2:** Technical parameters of tri-axial rotation platform.

Position Accuracy (°)	Rotation Rate Accuracy (°/s)	Maximum Rotation Rate (°/s)
1 × 10^−5^	1 × 10^−5^	±100

**Table 3:** Characteristics of tested IMU.

Characteristics	MTi-G	NAV440
Range (°/s)	−300~300	−400~400
Gyro bias instability (°/h)	3600	20
Gyro white noise (°/$\sqrt{h}$)	3.0	-

**Table 4:** RMS of navigation errors for conventional INS with MTi-G in static mode.

Latitude (m)	Longitude (m)	Height (m)
2412.3	1526.8	173.8
**Vn (m/s)**	**Ve (m/s)**	**Vu (m/s)**
33.2	20.5	1.2
**Pitch (°)**	**Roll (°)**	**Azimuth (°)**
1.83	1.32	2.31

**Table 5:** RMS of navigation errors for conventional INS with NAV440 in static mode.

Latitude (m)	Longitude (m)	Height (m)
829.1	2820.1	1033.0
**Vn (m/s)**	**Ve (m/s)**	**Vu (m/s)**
4.8	26.7	9.0
**Pitch (°)**	**Roll (°)**	**Azimuth (°)**
0.48	0.60	0.73

**Table 6:** Calibration errors caused by gyro noise for tested IMUs.

Rotation Rate (°/s)	MTi-G		NAV440	
	$\sigma_{b}$ (°/h)	$\sigma_{s}$ (ppm)	$\sigma_{b}$ (°/h)	$\sigma_{s}$ (ppm)
10	23.2	645.5	3.1	86.1
20		322.7		43.0
30		215.2		28.7
40		161.4		21.5
50		129.1		17.2
60		107.6		14.2
70		92.2		12.3
80		80.7		10.8

**Table 7:** Mean of gyro data after calibration for each individual rotary INS test.

Rotation Rate (°/s)	MTi-G			NAV440		
	X (°/s)	Y (°/s)	Z (°/s)	X (°/s)	Y (°/s)	Z (°/s)
10	−2.0 × 10^−3^	1.0 × 10^−2^	10.022	−2.0 × 10^−3^	−1.1 × 10^−2^	9.990
20	−7.1 × 10^−3^	1.4 × 10^−3^	20.020	9.8 × 10^−3^	−1.1 × 10^−3^	19.990
30	−1.4 × 10^−3^	1.9 × 10^−4^	30.024	7.9 × 10^−3^	9.6 × 10^−4^	29.996
40	−5.9 × 10^−4^	−7.8 × 10^−3^	40.008	8.7 × 10^−3^	2.2 × 10^−3^	39.999
50	−4.8 × 10^−3^	−2.5 × 10^−3^	50.001	2.0 × 10^−3^	−2.3 × 10^−3^	50.011
60	−1.5 × 10^−3^	1.7 × 10^−3^	60.008	4.5 × 10^−4^	−2.5 × 10^−3^	60.005
70	1.1 × 10^−2^	3.8 × 10^−3^	69.992	−2.9 × 10^−4^	−2.7 × 10^−3^	70.004
80	3.3 × 10^−3^	−1.6 × 10^−3^	79.988	−1.1 × 10^−3^	4.7 × 10^−3^	80.002
90	−8.3 × 10^−3^	−1.6 × 10^−3^	89.998	−3.3 × 10^-3^	5.2 × 10^−3^	90.005
100	9.4 × 10^−3^	3.1 × 10^−3^	99.993	−5.6 × 10^−3^	3.7 × 10^−3^	100.008
